# Upper-lower body super-sets *vs.* traditional sets for inducing chronic athletic performance improvements

**DOI:** 10.7717/peerj.14636

**Published:** 2023-02-21

**Authors:** Guillermo Peña García-Orea, David Rodríguez-Rosell, Ángel Ballester-Sánchez, Marzo Edir Da Silva-Grigoletto, Noelia Belando-Pedreño

**Affiliations:** 1 Department of Physical Activity and Sport, Universidad de Murcia, Murcia, Spain; 2 Physical Performance & Sports Research Center, Universidad Pablo de Olavide, Seville, Spain; 3 Faculty of Biomedical and Health Sciences, Universidad Europea de Madrid, Madrid, Spain; 4 Department of Physical Education, Federal University of Sergipe, São Cristóvão, Sergipe, Brazil

**Keywords:** Set configuration, Resistance training, Velocity-based training, Athletic performance, Muscle strength

## Abstract

**Background:**

To promote chronic adaptations, resistance training needs the manipulation of different variables, among them, the order of the exercises and sets. Specifically, for velocity-based training, paired exercises alternating upper and/or lower-body muscle groups appear to be a good choice to promote neuromuscular adaptations.

**Objective:**

This study aimed to compare the effect of two velocity-based training programs only differing in the set configuration on muscle strength, muscular endurance and jump performance.

**Methods:**

Moderately strength-trained men were allocated into a traditional (TS, n= 8) or alternating sets (AS, n= 9) configuration group to perform a 6-week velocity-based training program using the full squat (SQ) and bench press (BP) exercises. The TS group completed all sets of the full squat (SQ) exercise before performing the bench press (BP) sets, whereas the AS group completed the first set of each exercise in an alternating manner. Training frequency, relative load, number of sets, percentage of velocity loss (%VL) within the set and inter-set rest were matched for both groups. Countermovement jump height (CMJ), load (kg)-velocity relationship, predicted 1RM, and muscular endurance for each exercise were evaluated at pre- and post-training.

**Results:**

The TS and AS groups obtained similar and non-significant improvements in CMJ (3.01 ± 4.84% and 3.77 ± 6.12%, respectively). Both groups exhibited significant and similar increases in muscle strength variables in SQ (6.19–11.55% *vs.* 6.90-011.76%; *p* = 0.033–0.044, for TS and AS, respectively), BP (6.19–13.87% and 3.99–9.58%; *p* = 0.036–0.049, for TS and AS group, respectively), and muscular endurance in BP (7.29 ± 7.76% and 7.72 ± 9.73%; *p* = 0.033, for the TS and AS group, respectively). However, the AS group showed a greater improvement in muscular endurance in SQ than the TS group (10.19 ± 15.23% *vs.* 2.76 ± 7.39%; *p* = 0.047, respectively). Total training time per session was significantly shorter (*p* = 0.000) for AS compared to TS group.

**Conclusions:**

Training programs performing AS between SQ and BP exercises with moderate loads and %VL induce similar jump and strength improvements, but in a more time-efficient manner, than the traditional approach.

## Introduction

Configuration of the resistance training (RT) stimulus depends on the manipulation of different variables, such as exercise type and order, load magnitude, number of sets and repetitions, recovery time, and execution velocity (Kraemer & Ratamess, 2004; Bird, Tarpenning & Marino, 2005). Among these variables, the order of exercises and sets during the training session is of special relevance because this factor could influence the physiological response and the neuromuscular adaptations (Sforzo & Touey, 1996; Simão et al., 2012). In brief, training exercises could be configured using two procedures: (1) successively (*i.e.,* completing all training sets scheduled for a given exercise before performing the next training exercise), or (2) alternatively (*i.e.,* performing the first set of each exercise before continuing with the second set, and so on until completing all the scheduled sets). Regarding this, studies analyzing alternating exercise configurations have commonly used different paired set or super-set models, with agonist-antagonist (*e.g.*, bent-over row and bench press) and agonist-agonist pairings (*e.g.*, dumbbell bench press and barbell bench press) being the most investigated (Robbins et al., 2010a). However, paired exercises alternating upper- and lower-body limbs have rarely been considered (Weakley et al., 2020; Weakley et al., 2017).

The vast majority of studies addressing this issue have typically focused on analyzing the acute effect on physiological markers (Weakley et al., 2017; Paz et al., 2013; de Souza, Paz & Miranda, 2017; Miranda et al., 2018), rating of perceived exertion (Weakley et al., 2017; de Souza, Paz & Miranda, 2017), volume load (Robbins et al., 2010a; de Souza, Paz & Miranda, 2017; Robbins et al., 2010c; Robbins, Young & Behm, 2010; Paz et al., 2017), training efficiency (Robbins et al., 2010a; de Souza, Paz & Miranda, 2017; Robbins et al., 2010c), and variables related to mechanical and neuromuscular performance (Weakley et al., 2020; Weakley et al., 2017; Baker & Newton, 2005; Ciccone et al., 2014; Robbins et al., 2010b), highlighting potential adaptations. Although these investigations help to explain the differences between both configurations of sets, knowledge about the chronic effect is scarcer (Robbins et al., 2009; Merrigan, Jones & White, 2019). Accordingly, further studies comparing the effect of performing paired sets following a training intervention on neuromuscular function are needed.

On the other hand, most of the previous studies have used the maximum number of repetitions that can be performed with a given submaximal weight (*e.g.*, 8 RM, 10 RM) as a criterion to determine the relative load and prescribe RT, *i.e.,* performing each set at or close to muscle failure (Robbins et al., 2010a; Robbins et al., 2010c; Robbins et al., 2009). However, it has been observed that there is a large inter-participant variability (coefficient of variation ∼15–20%) in the maximum number of repetitions that can be completed to failure against different %1RM (Gonzalez-Badillo et al., 2017; Sanchez-Moreno et al., 2021; García-Ramos et al., 2018; García-Ramos et al., 2021). To solve the mentioned limitations, velocity-based training (VBT) has been recognized as an objective and valid methodology for training prescription and load monitoring during RT programs (Gonzalez-Badillo et al., 2017; Sanchez-Medina et al., 2014; Sanchez-Medina et al., 2017). VBT allows accurate and real-time relative load (%1RM) determination by measuring the fastest repetition of a set executed at maximal intended velocity (Sanchez-Medina et al., 2014; Sanchez-Medina et al., 2017; García-Ramos et al., 2021a). In addition, monitoring execution velocity loss (VL) during the set has been reported to be a reliable and good indicator of the neuromuscular fatigue that is been incurred and an accurate variable to prescribe training volume (Gonzalez-Badillo et al., 2017; Rodriguez-Rosell et al., 2020b; García-Ramos et al., 2018; Miras-Moreno, Pérez-Castilla & García-Ramos, 2022). Thus, instead of setting a fixed number of repetitions to be performed in the set, it is proposed to schedule the training volume by stopping the repetitions as soon as a certain magnitude (percentage) of VL has been reached (Gonzalez-Badillo et al., 2017).

Accordingly, taking into account the above considerations, it is unknown whether an alternating set configuration implying opposite limbs (*i.e.,* upper- and lower-body limbs) may be more, equally, or less effective for this purpose than a traditional approach, but with the advantage of saving considerable training time during the session. Thus, the research presented in this manuscript aimed to compare the effects of traditional *vs.* alternating set configuration, both using VBT methodology, on athletic performance following a training period. In this way, we hypothesize that performing both set configurations using full squat and bench press exercises will produce similar effects on athletic performance if a moderate degree of fatigue in the set is induced.

## Materials & Methods

### Experimental design

**Figure 1:**
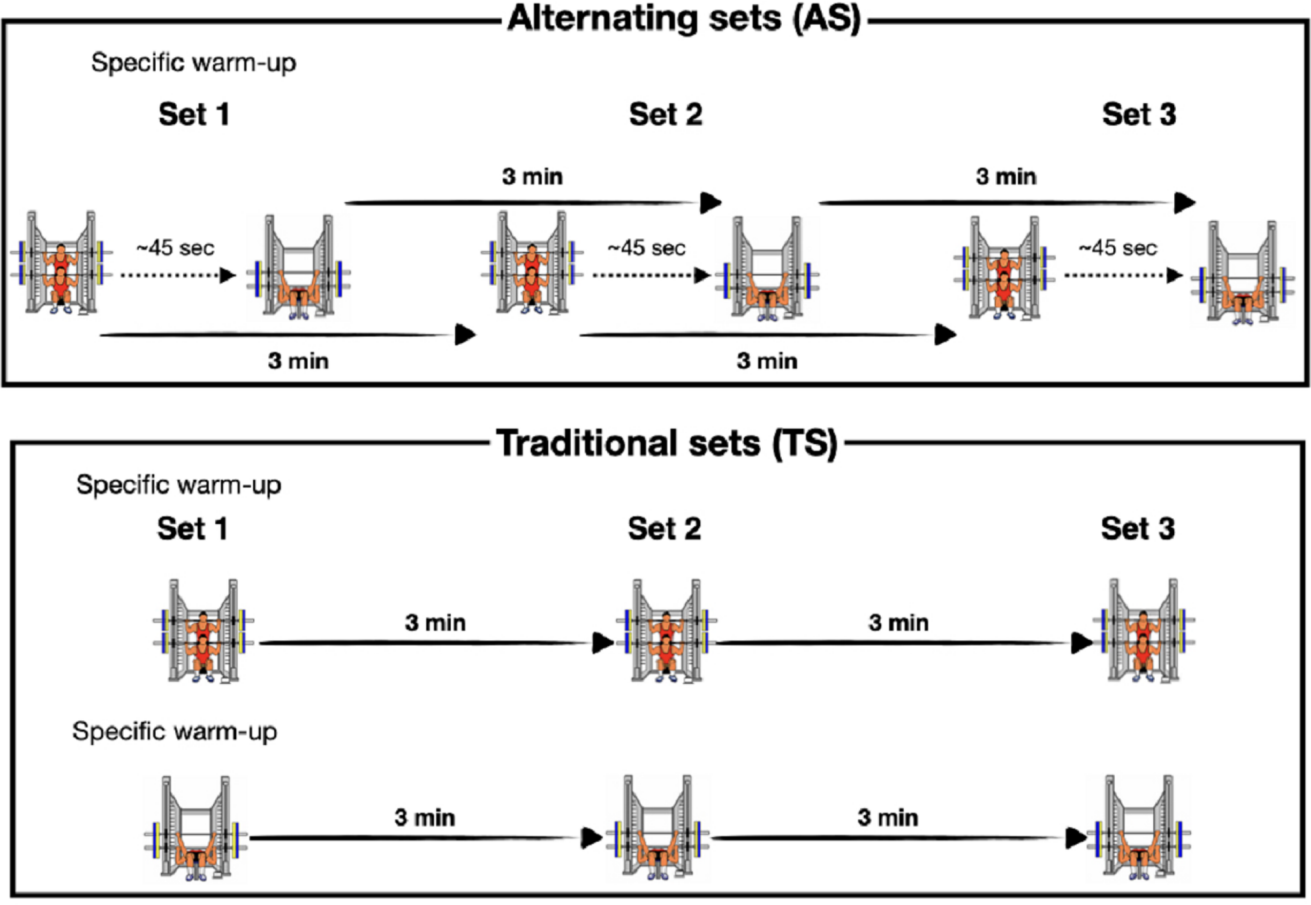
Experimental design.

We conducted a longitudinal and quasi-experimental study to compare the changes in athletic performance following a VBT program. Two experimental groups differed in the arrangement in which the scheduled sets (three sets per exercise) of the full squat (SQ) and bench press (BP) exercises were performed. The traditional sets (TS) group completed all sets of the SQ exercise before performing the BP sets, whereas the alternating sets (AS) group completed the first set of each exercise in an alternating manner (Fig. 1). The same inter-set rest interval was established for each exercise (3 min). However, a 45 s time interval between the SQ and BP in each set was established for the AS group. Participants underwent a two weeks familiarization period prior to the intervention to improve the execution technique of the SQ and PB exercise and countermovement jump (CMJ).

For the study, both groups trained twice a week for six weeks using the same: (1) training exercises and order (SQ followed by BP); (2) relative intensity (55–70% 1RM); (3) percentage of velocity loss (%VL) in each training set (15% and 20% for SQ and BP, respectively); (4) number of sets; (5) inter-set recovery and rest periods between sessions (72–96 h). All sessions were conducted under the supervision of experienced researchers at the same time slot for each participant and under similar temperature and humidity conditions (22−25 ° C and ∼60%, respectively). Participants were asked to abstain from any strenuous physical exercise for the duration of the study. All participants were assessed with the same testing procedure (described below) before (pre-) and after (post-) the 6-week training intervention using a battery of tests. The time elapsed between the Pre-test and the start of the training intervention was 72–96 h. The same time was set between the end of the training and the post-test.

### Participants

Seventeen moderately strength-trained (predicted SQ 1RM: 93.6 ± 19.1 kg, predicted BP 1RM: 71.9 ± 12.4 kg) young men (age: 23.9 ± 5.3 years, body mass: 72.9 ± 10 kg, height: 1.72 ± 0.08 m) participated in the study. Participants were matched according to their relative strength (predicted 1RM/body mass) in the SQ and BP exercises and randomly allocated into two groups: traditional sets (TS, *n* = 8) or alternating sets (AS, *n* = 9) group. The study was conducted following the ethical standards of the Declaration of Helsinki. The University of Murcia granted Ethical approval to carry out the study within its facilities. 1862/2018. All participants signed a written consent form after being informed of the risks, purpose, and experimental procedures of the study.

### Testing procedures

We measured the physical performance through a battery of tests performed in a single session and a fixed sequence. The sequence was: (1) CMJ; (2) progressive loading test in SQ; (3) fatigue test in SQ; (4) progressive loading test in BP; (5) fatigue test in BP. Before performing the first test, participants accomplished a general standardized warm-up protocol for 10 min, consisting of jogging, joint mobilization exercises, and dynamic stretching.

#### Countermovement jump test

For the performance of this test, the subjects stand in an upright position with their hands placed on the hips to avoid arm swings. A fast downward movement (knee flexion), with depth self-selected by the subject, was immediately followed by a fast upward vertical movement (knee extension) to jump as high as possible, all in one sequence. We instructed the participants to keep their knees straight during the flight phase of the jump and to land in an upright position. Each subject performed five maximal CMJs, with 40 s rest in-between. We discard the highest and lowest values and use the resulting average of the three remaining trials for analysis (Rodriguez-Rosell et al., 2020b). Jump height was estimated by an infrared timing device that measures flight time (Optojump Next; Microgate, Bolzano, Italy). The specific warm-up consisted of two sets of 6 repetitions at a moderate velocity of bodyweight squats (2 min rest interval), five CMJs at progressive effort (30 s rest), and three CMJs at maximal effort (45 s rest).

#### Progressive loading test

We conducted this test to determine the individual load(kg)-velocity relationship and estimation of the 1RM value in the SQ and BP exercise. A detailed description of this testing protocol and execution technique for both exercises has been provided elsewhere (Sanchez-Medina et al., 2017; Pallares et al., 2014; Sanchez-Medina, Perez & Gonzalez-Badillo, 2010). The initial load was set for all participants at 25 and 20 kg for SQ and BP, respectively. The load was gradually increased by 10 kg for each set until the mean propulsive velocity (MPV) was equal to or below the value corresponding to ∼80% 1RM in each exercise (SQ: ≤0.68 m s^−1^, BP: ≤0.47 m s^−1^) (Sanchez-Medina et al., 2017; Gonzalez-Badillo & Sanchez-Medina, 2010). We used an identical specific warm-up (explained later) and progression of absolute loads in pre- and post- for each subject. We considered only the best repetition (correctly executed) against each absolute load (kg), according to the criterion of fastest MPV, for subsequent analysis (García-Ramos et al., 2021). The 1RM (kg) value was estimated individually from the MPV reached against the heaviest load (kg) used in each test according to the following equations: 100 × kg/(−5.961 × MPV^2^) − (50.71 × MPV) + 117, for the SQ;22 and 100 × kg/(8.4326 × MPV^2^) − (73.501 × MPV) + 112.33, for the BP (Gonzalez-Badillo & Sanchez-Medina, 2010). As previously used in other studies (González-Badillo et al., 2014), three other variables were used to analyze the extent to which the two training conditions (TS and AS) affected different parts of the load(kg)-velocity relationship: (a) average MPV against all absolute loads common to pre- and post-tests (AV); (b) average MPV against absolute loads common to both tests that were lifted faster than 1.00 m s^−1^ (AV>1) or 0.80 m s^−1^ (AV>0.8) for SQ and BP, respectively; or (c) average MPV attained against absolute loads common to both tests that were lifted slower than 1.00 m s^−1^ (AV<1) or 0.80 m s^−1^ (AV<0.8) for SQ and BP, respectively. These other variables have been shown as highly reliable (Pallares et al., 2014), and provide a much more comprehensive analysis of training-induced changes across the load-velocity spectrum rather than simply focusing on a 1RM value (Pareja-Blanco et al., 2014). Specific warm-up consisted of two sets of five and three repetitions (2 min inter-set rest) with loads of 20 and 25 kg at high and maximal intended velocity, respectively, was performed before each progressive loading test. The progressive loading test in BP was performed following a five min rest after the fatigue test in the SQ exercise (explained later).

#### Fatigue test in the SQ and BP exercise

This test was performed 5 min after participants finished the progressive loading test of each exercise. Both pre-tests were performed against an absolute load that elicited a MPV value corresponding to ∼60% 1RM (±2%) in each exercise. Thus, before starting the test, adjustments in the load (kg) to be lifted were made so that the velocity of the first repetition matched the required target MPV (*i.e.,* SQ: 1.00 ±  0.03 m s^−1^; BP: 0.78 ± 0.03 m s^−1^). For both exercises, participants were required to lift the barbell as fast as possible during the concentric phase and complete 12 repetitions. To estimate changes in muscular endurance, both fatigue tests (pre- and post-) were performed against the same absolute loads, established individually in each pre-test (SQ and BP), and completed the same number of repetitions. Performance was determined by the average MPV in the set of 12 repetitions.

### Resistance training program

Preceding all training sessions, participants for both groups conducted a general standardized warm-up (explained above) and the same specific warm-up consisting of three sets of eight, five, and two repetitions (2 min inter-set rest) with loads of 20 kg, 25 kg and the proposed absolute load that best matched the scheduled target MPV, at moderate, high, and maximal intended velocity, respectively. Descriptive characteristics of the RT performed by both groups are reported in Table 1 (SQ exercise) and Table 2 (BP exercise). Following the initial evaluation, all participants conducted a 6-week VBT program involving two sessions per week (12 total sessions) with 72–96 h of rest between sessions, performing the SQ and BP exercises in the same order. Except for the set structuring between exercises (*i.e.,* TS *vs.* AS), training variables (fastest MPV of the first set, %VL within the set for each exercise, number of sets per exercise, recovery time between sets, and number of sessions using each relative load) were the same for the two experimental conditions throughout the training intervention. Individualization of the training relative load (%1RM) was determined from the general load-velocity relationship for SQ22 and BP (Gonzalez-Badillo & Sanchez-Medina, 2010). by means of the MPV of the fastest repetition of the first set, and the volume (number of repetitions) to perform in each exercise set was objectively determined utilizing the %VL attained in the set (Rodriguez-Rosell et al., 2020), as previously described in Peña García-Orea et al. (2022), regardless of the number of repetitions completed by each subject (Rodriguez-Rosell et al., 2020b; Pareja-Blanco et al., 2017a; Sanchez-Medina & Gonzalez-Badillo, 2011). Fixed moderate magnitudes of intra-set VL for each exercise were established for all sessions to provide an analogous level of fatigue across the participants at the end of each set (Gonzalez-Badillo et al., 2017). Different %VL between exercises (15% *vs.* 20% for SQ and BP, respectively) were used to match the same percentage of repetitions per set completed concerning the maximum possible repetitions against each relative load (Rodriguez-Rosell et al., 2020).

**Table 1:** Descriptive characteristics of the velocity-based squat training program performed by both experimental groups.

Scheduled	Session 1	Session 2	Session 3	Session 4	Session 5	Session 6	Session 7	Session 8	Session 9	Session 10	Session 11	Session 12	Average
Target MPV (m s^−1^)	1.07	1.07	1.07	1.00	1.00	1.00	0.92	0.92	0.92	0.84	0.84	0.84	0.96
	(∼55% 1RM)	(∼55% 1RM)	(∼55% 1RM)	(∼60% 1RM)	(∼60% 1RM)	(∼60% 1RM)	(∼65% 1RM)	(∼65% 1RM)	(∼65% 1RM)	(∼70% 1RM)	(∼70% 1RM)	(∼70% 1RM)	(62.5%)
Sets x VL (%)	3 × 15%	3 × 15%	3 × 15%	3 × 15%	3 × 15%	3 × 15%	3 × 15%	3 × 15%	3 × 15%	3 × 15%	3 × 15%	3 × 15%	3 × 15%
Actually performed MPV_BEST_ (m s^−1^)													Average
TS	1.08 ± 0.03	1.08 ± 0.02	1.08 ± 0.02	1.02 ± 0.02	1.01 ± 0.02	1.00 ± 0.03	0.93 ± 0.02	0.91 ± 0.02	0.91 ± 0.02	0.84 ± 0.01	0.83 ± 0.02	0.84 ± 0.02	0.96 ± 0.02
	(∼55.4% 1RM)	(∼55.3% 1RM)	(∼55.3% 1RM)	(∼59.5% 1RM)	(∼59.6% 1RM)	(∼60.3% 1RM)	(∼64.9% 1RM)	(∼65.6% 1RM)	(∼66.0% 1RM)	(∼70.3% 1RM)	(∼71.0% 1RM)	(∼70.1% 1RM)	(62.8% 1RM)
AS	1.08 ± 0.02	1.07 ± 0.02	1.08 ± 0.03	1.00 ± 0.02	1.00 ± 0.03	1.01 ± 0.03	0.92 ± 0.02	0.93 ± 0.01	0.91 ± 0.02	0.84 ± 0.02	0.84 ± 0.02	0.84 ± 0.02	0.96 ± 0.02
	(∼55.0% 1RM)	(∼55.7% 1RM)	(∼55.5% 1RM)	(∼60.1% 1RM)	(∼60.5% 1RM)	(∼59.7% 1RM)	(∼65.1% 1RM)	(∼64.8% 1RM)	(∼65.7% 1RM)	(∼69.9% 1RM)	(∼70.3% 1RM)	(∼70.2% 1RM)	(62.7% 1RM)
Intra-set VL (%)													Average
TS	19.1 ± 2.4	17.8 ± 2.4	16.8 ± 1.3	18.5 ± 1.0	17.8 ± 2.4	17.3 ± 2.1	16.2 ± 1.5	17.0 ± 2.5	16.0 ± 0.9	16.8 ± 3.0	16.1 ± 1.5	16.2 ± 1.2	17.1 ± 1.8
AS	19.3 ± 2.3	19.2 ± 3.0	17.8 ± 1.2	18.0 ± 2.1	18.4 ± 2.0	17.6 ± 2.4	17.1 ± 2.3	16.7 ± 1.7	15.5 ± 1.1	16.0 ± 1.2	16.3 ± 1.7	16.3 ± 1.2	17.3 ± 1.9
Reps per set (#)													Average
TS	11.2 ± 3.8	10.8 ± 3.5	10.9 ± 2.2	9.0 ± 2.7	8.2 ± 3.2	8.5 ± 3.9	7.0 ± 2.3	6.5 ± 2.0	6.2 ± 3.0	5.1 ± 1.4	5.7 ± 1.8	4.7 ± 1.1	93.8 ± 2.3
AS	8.3 ± 2.3	8.7 ± 2.1	8.0 ± 1.7	7.4 ± 2.4	6.8 ± 1.7	6.6 ± 2.8**	5.4 ± 1.3*	5.5 ± 1.4	5.4 ± 1.7	4.3 ± 1.1*	4.0 ± 1.2	4.2 ± 1.2	74.6 ± 1.6^**^

**Notes.**

Data are mean ± SD. MPV: mean propulsive velocity attained against the intended load (%1RM); VL: Velocity loss; Reps per set: number of repetitions performed; MPVBEST: mean propulsive velocity of the fastest (usually first) repetition in the first set.

The actual velocity losses and repetitions per set reported are the mean of the three sets.

TS Traditional set group AS Alternating set group

Statistically significant differences with respect to:

* TS group (*p* <  0.05).

** TS group (*p* < 0.01).

Statistical method used: Student’s *t*-test.

**Table 2:** Descriptive characteristics of the velocity-based bench press training program performed by both experimental groups.

Scheduled	Session 1	Session 2	Session 3	Session 4	Session 5	Session 6	Session 7	Session 8	Session 9	Session 10	Session 11	Session 12	Average
Target MPV (m s^−1^)	0.87	0.87	0.87	0.78	0.78	0.78	0.70	0.70	0.70	0.62	0.62	0.62	0.74
	(∼55% 1RM)	(∼55% 1RM)	(∼55% 1RM)	(∼60% 1RM)	(∼60% 1RM)	(∼60% 1RM)	(∼65% 1RM)	(∼65% 1RM)	(∼65% 1RM)	(∼70% 1RM)	(∼70% 1RM)	(∼70% 1RM)	(62.5%)
Sets × VL (%)	3 × 20%	3 × 20%	3 × 20%	3 × 20%	3 × 20%	3 × 20%	3 × 20%	3 × 20%	3 × 20%	3 × 20%	3 × 20%	3 × 20%	3 × 20%
Actually performed MPV_BEST_ (m s^−1^)													Average
TS	0.88 ± 0.01	0.87 ± 0.02	0.87 ± 0.02	0.79 ± 0.02	0.78 ± 0.02	0.78 ± 0.03	0.69 ± 0.01^*^	0.69 ± 0.01	0.69 ± 0.02	0.61 ± 0.03	0.62 ± 0.02	0.62 ± 0.02	0.74 ± 0.02
	(∼54.4% 1RM)	(∼54.6% 1RM)	(∼55.0% 1RM)	(∼59.4% 1RM)	(∼59.9% 1RM)	(∼60.3% 1RM)	(∼65.9% 1RM)	(∼65.4% 1RM)	(∼65.5% 1RM)	(∼70.5% 1RM)	(∼70.1% 1RM)	(∼70.3% 1RM)	(62.6%)
AS	0.87 ± 0.01	0.88 ± 0.02	0.86 ± 0.02	0.78 ± 0.02	0.77 ± 0.03	0.78 ± 0.02	0.71 ± 0.02^*^	0.70 ± 0.02	0.70 ± 0.03	0.62 ± 0.02	0.62 ± 0.02	0.62 ± 0.02	0.74 ± 0.02
	(∼55.0% 1RM)	(∼54.2% 1RM)	(∼55.4% 1RM)	(∼60.4% 1RM)	(∼60.9% 1RM)	(∼60.0% 1RM)	(∼64.3% 1RM)	(∼64.9% 1RM)	(∼64.9% 1RM)	(∼70.1% 1RM)	(∼70.1% 1RM)	(∼70.2% 1RM)	(62.5%)
Intra-set VL (%)													Average
TS	22.2 ± 2.2	22.1 ± 0.9	23.2 ± 1.6	23.1 ± 1.9	22.2 ± 2.3	21.8 ± 1.8	21.6 ± 2.1	20.9 ± 2.1	20.4 ± 1.2	20.3 ± 1.2	21.3 ± 1.9	21.1 ± 2.3	21.7 ± 1.8
AS	21.8 ± 1.3	22.6 ± 1.2	22.2 ± 2.5	22.5 ± 2.1	23.2 ± 1.4	22.7 ± 1.9	22.4 ± 2.4^*^	21.2 ± 1.0	20.5 ± 1.9	21.2 ± 1.6	20.7 ± 2.3	20.5 ± 1.8	21.8 ± 1.8
Reps per set (#)													Total
TS	9.5 ± 2.2	10.5 ± 3.7	9.7 ± 3.7	8.0 ± 2.9	7.1 ± 2.0	6.6 ± 2.2	5.6 ± 1.5	5.4 ± 1.3	5.5 ± 1.4	4.3 ± 1.3	4.2 ± 1.2	4.2 ± 0.7	80.7 ± 2.2
AS	9.4 ± 2.0	9.1 ± 1.2^*^	8.4 ± 1.5^*^	7.5 ± 1.1	7.1 ± 1.2	7.8 ± 1.7	6.1 ± 1.3	5.3 ± 1.0	5.6 ± 1.2	4.4 ± 0.6	4.4 ± 0.8	4.3 ± 0.8	79.3 ± 1.9

**Notes.**

Data are mean ± SD.

MPV mean propulsive velocity attained against the intended load (%1RM) VL Velocity loss Reps per set number of repetitions performed MPV_BEST_ mean propulsive velocity of the fastest (usually first) repetition in the first set

The actual velocity losses and repetitions per set reported are the mean of the three sets.

TS Traditional set group AS Alternating set group. Statistically significant differences with respect to

* TS group (*p* < 0.05).

Statistical method used: Student’s *t*-test.

During each training session, participants received immediate velocity feedback while being encouraged to perform each repetition at maximal intended velocity (*i.e.,* as fast as possible).

### Equipment and data acquisition

All testing and training sessions were performed on the same Smith machine (Multipower, Technogym). A linear velocity transducer (T-Force Dynamic Measurement System; Ergotech Consulting Ltd, Murcia, Spain) and its associated software (version 3.70) were used for recording and saving velocity and displacement values of every repetition during all sessions. A complete analysis of the T-Force System linear velocity transducer reliability is reported elsewhere (Courel-Ibanez et al., 2019; Sanchez-Medina & Gonzalez-Badillo, 2011; Martinez-Cava et al., 2020). All velocity measures reported in this study corresponded to the MPV of each repetition (Sanchez-Medina, Perez & Gonzalez-Badillo, 2010).

### Statistical analyses

Shapiro–Wilk test was used to verify the normality of distribution of the variables at Pre-. Levene’s test was used to confirm homogeneity of variance across groups (TS *vs.* AS). The training-related effects were assessed using a two (group: TS *vs.* AS) × two (time: Pre- *vs.* Post-) factorial ANOVA. Similarly, differences in training variables (MPV of the first repetition, intra-set %VL, and the number of repetitions performed at different velocity ranges) between training conditions were examined using a Student’s *t*-test for independent variables. The intra-group effect size (ES) was calculated using Hedge’s g on the pooled SD (Hedges & Olkin, 1985), considering small (<0.20), medium (0.21−0.50), large (0.51−0.80), and very large (0.81−1.30) values. Intra-group percent change was also estimated for each dependent variable between both time points (Pre- *vs.* Post-). We considered a statistical significance when *p* < 0.05. Null hypothesis tests were performed using SPSS software version 25.0 (SPSS, Chicago, IL, USA).

## Results

All participants considered for data analysis completed 100% of the scheduled training sessions. Data for all variables analyzed were homogeneous and normally distributed. We did not find significant differences between groups (TS *vs.* AS) at Pre- for any of the variables analyzed.

### Training program

Tables 1 and 2 show that MPV of the fastest repetition of the first set (*i.e.,* relative load) and the actual average %VL over the three sets closely concurred those scheduled for each exercise in the training sessions. Differences between groups in the average %VL incurred over the three sets were non-significant in the SQ (17.1 ±  1.8% and 17.3 ± 1.9% for TS and AS group, respectively) or the BP exercise (21.7 ± 1.8% and 21.8 ± 1.8% for TS and AS group, respectively).

Participants of both experimental groups trained at the same average MPV (0.96 ± 0.02 m s^−1^ *vs.* 0.96 ± 0.0.02 m s^−1^ in the SQ and 0.74 ± 0.02 m s^−1^ *vs.* 0.74 ± 0.02 m s^−1^ in the BP exercise, respectively) during the training period. Additionally, no significant differences between groups were observed for the fastest MPV of the first set in any session and exercise, except for session 7 in the BP exercise (Tables 1 and 2).

Comparisons of the average total number of repetitions performed during the whole 6-week period in the SQ exercise revealed that participants of the TS group performed significantly (*p* = 0.029) more repetitions (93.8 ± 2.3) than those completed by the AS group (74.6 ± 1.6) (Table 1). However, both experimental groups completed a very similar total number of repetitions (80.7 ± 2.2 *vs.* 79.3 ± 1.9 for TS and AS groups, respectively) in the BP exercise during the same training period (Table 2).

The analysis of number of repetitions performed at different velocity ranges in the SQ exercise (Table 3) revealed that a slightly greater number of repetitions at faster velocities and a higher number of total repetitions were completed by the TS group than the AS group (290.6 ± 91.5 *vs.* 222.8 ± 47.8 for the TS and AS group, respectively) during the entire training intervention. For the BP exercise, there were no significant differences between training groups in the number of repetitions completed in any velocity range, except for the range of 0.8−0.9 m s^−1^ (Table 4). In addition, both groups completed a similar number of total repetitions (228.1 ± 72.2 *vs.* 224.4 ± 33.2 for TS and AS groups, respectively) during the training period. Finally, between-group differences in total training time completed per session, including the standardized warm-up, revealed significantly shorter (*p* = 0.000) total workout duration for the AS group (23.3 min ± 2.2 min) than for the TS group (42.2 min ± 3.1 min).

**Table 3:** Number of repetitions performed in each velocity range and total number of repetitions completed by both training groups in the full squat exercise.

MPV (m s^−^^1^)	TS	AS
<0.3	0.0 ± 0.0	0.0 ± 0.0
>0.3–0.4	0.0 ± 0.0	0.0 ± 0.0
>0.4–0.5	0.0 ± 0.0	0.0 ± 0.0
>0.5–0.6	0.3 ± 0.5	0.2 ± 0.7
>0.6–0.7	8.6 ± 5.0	8.1 ± 3.7
>0.7–0.8	45.1 ± 7.8	44.2 ± 11.1
>0.8–0.9	87.0 ± 37.3	75.8 ± 24.5
>0.9–1.0	100.4 ± 45.4	68.4 ± 15.1^*^
>1.0–1.1	47.9 ± 24.6	25.4 ± 9.5^*^
>1.1	2.0 ± 4.6	0.9 ± 1.4
Total reps	290.6 ± 91.5	222.8 ± 47.8^*^

**Notes.**

Data are mean ± SD. The experimental groups trained with different set configurations. TS (*n* = 8), AS (*n* = 9). Statistically significant differences with respect to TS group.

* (*p* < 0.05). Statistical method used: Student’s *t*-test.

Abbreviations TS Traditional sets experimental group AS Alternating sets experimental group MPV mean propulsive velocity Reps number of repetitions performed

**Table 4:** Number of repetitions performed in each velocity range and total number of repetitions completed by both training groups in the bench press exercise.

MPV (m s^−^^1^)	TS	AS
<0.3	0.0 ± 0.0	0.0 ± 0.0
>0.3–0.4	0.0 ± 0.0	0.5 ± 1.3
>0.4–0.5	9.0 ± 3.7	9.1 ± 3.4
>0.5–0.6	45.1 ± 11.1	51.9 ± 10.6
>0.6–0.7	76.1 ± 24.5	83.9 ± 17.4
>0.7–0.8	76.0 ± 33.7	67.0 ± 9.8
>0.8–0.9	35.6 ± 12.9	24.9 ± 7.1^*^
>0.9–1.0	0.3 ± 0.7	1.0 ± 2.0
>1.0–1.1	0.0 ± 0.0	0.0 ± 0.0
>1.1	0.0 ± 0.0	0.0 ± 0.0
Total reps	228.1 ± 72.2	224.4 ± 33.2

**Notes.**

Data are mean ± SD. The experimental groups trained with different set configurations: TS (*n* = 8), AS (*n* = 9). Statistically significant differences with respect to TS group.

* *p* < 0.05).

Statistical method used: Student’s *t*-test.

Abbreviations TS Traditional sets experimental group AS Alternating sets experimental group MPV mean propulsive velocity Reps number of repetitions performed

### Jump and strength variables

Changes in the selected neuromuscular (athletic) performance variables for SQ and BP exercises are displayed in Tables 5 and 6, respectively. A significant “time × group” interaction (*p* = 0.047) was only found for the fatigue test in the SQ exercise, with AS group showing greater increments compared to TS (ES: 0.86 *vs.* 0.55; Δ: 10.19 ± 15.23% *vs.* 2.76 ± 7.39%, respectively). No significant differences were observed between both experimental groups in any other variable analyzed in each exercise (predicted 1RM, AV, AV ≥1, AV<1, AV ≥0.8, AV<0.8).

**Table 5:** Changes in selected neuromuscular performance variables from pre- to posttraining for each group in the countermovement jump and squat exercises.

	TS				AS			
	Pre	Post	Δ (%)	ES	Pre	Post	Δ (%)	ES
CMJ (cm)	37.48 ± 6.22	38.61 ± 5.93	3.01 ± 4.84	0.18	37.37 ± 6.22	38.78 ± 5.52^*^	3.77 ± 6.12	0.23
1RM (kg)	89.00 ± 10.39	95.88 ± 11.36^**^	7.72 ± 5.35	0.60	97.88 ± 27.08	104.63 ± 26.86	6.90 ± 10.63	0.24
AV (m s ^−1^)	0.93 ± 0.06	1.01 ± 0.09^*^	8.48 ± 7.99	0.99	0.96 ± 0.10	1.06 ± 0.10^*^	10.55 ± 12.08	0.95
AV ≥1 (m s ^−1^)	1.14 ± 0.06	1.21 ± 0.10^*^	6.19 ± 5.80	0.80	1.17 ± 0.11	1.29 ± 0.08^*^	9.94 ± 10.57	1.19
AV<1 (m s ^−1^)	0.78 ± 0.04	0.87 ± 0.10^*^	11.55 ± 9.00	1.12	0.80 ± 0.03	0.90 ± 0.11^*^	11.76 ± 12.95	1.18
Fatigue test AV (m s ^−1^)	0.91 ± 0.02	0.94 ± 0.07	2.76 ± 7.39	0.55	0.87 ± 0.02	0.96 ± 0.14	10.19 ± 15.23^#^	0.86

**Notes.**

Data are mean ± SD. The experimental groups trained with different set configurations: TS (*n* = 8), AS (*n* = 9). Statistically significant ”time x group” interaction.

# *p* < 0.05. Statistically significant differences with respect to TS #*p* < 0.05. Intra-group significant differences from pre- to post-training.

* *p* < 0.05.

** *p* < 0.01. Statistical method used: factorial ANOVA.

Abbreviations TS Traditional sets experimental group AS Alternating sets experimental group Pre initial assessment Post final assessment ES intra-group effect size (Hedge’s d values: small: <0.20; medium: 0.21–0.50; large: 0.51–0.80; very large: 0.81–1.30). 1RM estimated one-repetition maximum in the full squat exercise CMJ countermovement jump height AV average MPV attained against all absolute loads common to pre-and post-tests in the squat progressive loading test AV ≥1 average MPV attained against common loads that were lifted equal or faster than 1.00 m s ^−1^ in the squat progressive loading test AV <1 average MPV against common loads lifted slower than 1.00 m s ^−1^ in the squat progressive loading test fatigue test AV average MPV attained against the same absolute load and number of reps common to pre- and post-tests in the squat fatigue test

**Table 6:** Changes in selected neuromuscular performance variables from pre- to posttraining for each group in the bench press exercise.

	TS				AS			
	Pre	Post	△ (%)	ES	Pre	Post	△ (%)	ES
1RM (kg)	71.00 ± 13.19	76.00 ± 13.06^*^	7.04 ± 6.38	0.36	72.70 ± 12.41	75.60 ± 12.62^*^	3.99 ± 3.58	0.22
AV (m s ^−1^)	0.78 ± 0.04	0.85 ± 0.05^**^	9.02 ± 5.75	1.47	0.79 ± 0.05	0.84 ± 0.06^**^	7.19 ± 6.16	0.86
AV ≥0.8 (m s ^−1^)	1.06 ± 0.05	1.12 ± 0.05^*^	6.19 ± 6.26	1.14	1.06 ± 0.07	1.11 ± 0.10^*^	4.74 ± 5.33	0.55
AV<0.8 (m s ^−1^)	0.53 ± 0.05	0.61 ± 0.06^**^	13.87 ± 9.40	1.38	0.57 ± 0.04	0.62 ± 0.05^**^	9.58 ± 8.00	1.05
Fatigue test AV (m s ^−1^)	0.65 ± 0.05	0.70 ± 0.08^*^	7.29 ± 7.76	0.71	0.64 ± 0.03	0.69 ± 0.06^*^	7.72 ± 9.73	1.00

**Notes.**

Data are mean ± SD. The experimental groups trained with different set configurations: TS (*n* = 8), AS (*n* = 9). Statistically significant “time × group” interaction.

# *p* < 0.05. Statistically significant differences with respect to TS #*p* < 0.05. Intra-group significant differences from pre- to post-training.

* *p* < 0.05.

** *p* < 0.01. Statistical method used: factorial ANOVA.

Abbreviations TS Traditional sets experimental group AS Alternating sets experimental group Pre initial assessment Post final assessment ES intra-group effect size (Hedge’s d values: small: <0.20; medium: 0.21–0.50; large: 0.51–0.80; very large: 0.81–1.30) 1RM estimated one-repetition maximum in the bench press exercise AV average MPV attained against all absolute loads common to pre-and post-tests in the bench press progressive loading test AV ≥0.8 average MPV attained against common loads that were lifted equal or faster than 0.80 m s ^−1^ in the bench press progressive loading test AV <0.8 average MPV against common loads lifted slower than 0.80 m s ^−1^ in the bench press progressive loading test fatigue test AV average MPV attained against the same absolute load and number of reps common to pre- and post-tests in the bench press fatigue test

**Figure 2:**
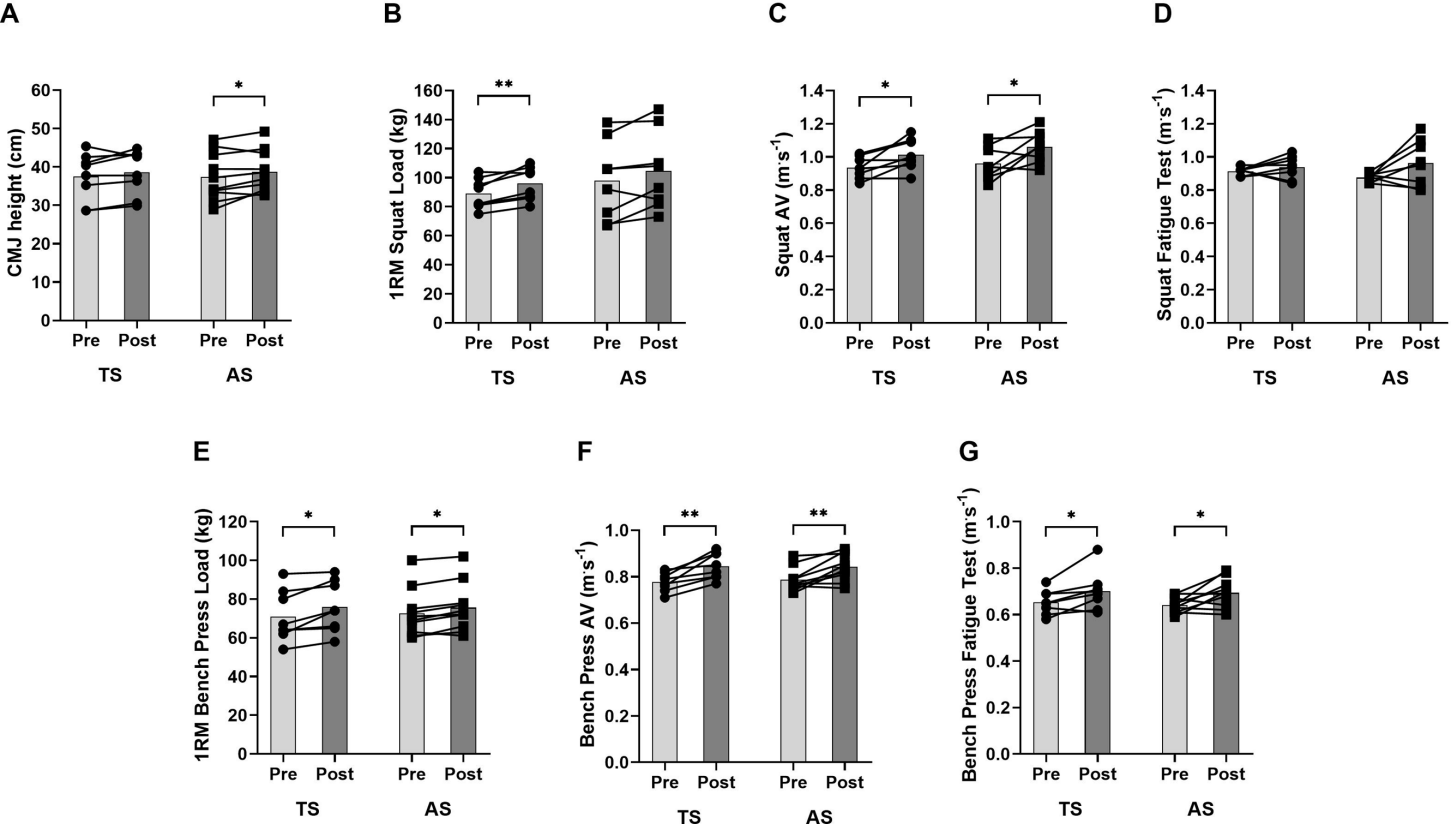
Changes in jump and strength variables following 6-week of velocity-based training. (A) CMJ height; (B) estimated squat 1RM (kg); (C) average velocity attained against loads in the squat progressive loading test; (D) fatigue test in the squat exercise; (E) estimated bench press 1RM (kg); (F) average velocity attained against loads in the bench press progressive loading test; (G) average velocity in the fatigue test in the bench press exercise. TS, Traditional sets group (*n* = 8); AS, Alternating sets group (*n* = 9). Statistically significant “time × group” interaction: #*p* < 0.05. Statistically significant differences with respect to TS #*p* < 0.05. Intra-group significant differences from pre- to post-training: ^∗^*p* < 0.05, ^∗∗^*p* < 0.01.

Both groups exhibited small and similar increments in CMJ from pre- to post-training (ES: 0.23 and 0.18 for AS and TS groups, respectively), although only the AS group exhibited significant differences (*p* = 0.47) (Table 5 and Fig. 2A). Both the TS and AS groups showed significant large and very large pre-post increments in most strength variables obtained from the load-velocity relationship in the SQ exercise (6.19–11.55% and 6.90–11.76%; ES: 0.60−1.12 and 0.24−1.19; *p* = 0.033−0.044, respectively) after the training period (Table 5 and Figs. 2C, 2D). None of the two experimental groups showed significant differences in the fatigue test in the SQ exercise (Table 5 and Fig. 2D). With respect to the upper-body, TS and AS group revealed significant medium and very large pre-post improvements in all strength variables assessed in the BP exercise (6.19–13.87% and 3.99−9.58%; ES: 0.36−1.47 and 0.22−1.05; *p* = 0.036−0.049, respectively) and in the fatigue test (7.29 ± 7.76% and 7.72 ± 9.73%; ES: 0.71 and 1.00; *p* = 0.033, respectively) (Table 6 and Figs. 2E, 2F, 2G).

## Discussion

The purpose of the present study was to compare the effect of two VBT programs with different set configurations (TS *vs.* AS) on athletic performance. To the best of our knowledge, this is the first study comparing traditional *vs.* alternating sets configurations: (1) using paired exercises involving upper- and lower-limbs muscles groups (SQ and BP exercises) through longitudinal research with identical training protocol, (2) adjusting and matching the relative load (%1RM) and training volume (%VL in the set) in each session for all participants using a VBT methodology, and (3) analyzing the training effect with a variety of physical performance variables. The main finding of the current investigation was that the AS group obtained similar improvements in CMJ, strength, and muscular endurance in SQ and BP to the TS group. Consequently, our results suggest that the AS configuration is a more time-efficient training strategy because it roughly halves that for the TS condition. This training strategy allows to optimize training time without compromising strength gains, at least when both combined exercises (*i.e.,* SQ and BP) are performed with moderate relative loads (55–70% 1RM) and attaining a low degree of fatigue at the end of each set (15–20% VL).

The RT program applied in this study resulted in significant improvements in most strength variables assessed in the SQ and BP exercise for both experimental groups (Tables 5 and 6). Although no statistically significant differences between groups were observed, the TS group showed greater pre-post percent changes and ES in the strength variables evaluated during the BP exercise than the AS group (Table 6). These results were obtained even though both groups completed a similar total number of repetitions during the training program (Tables 2 and 4). This slight tendency to better results in strength gains during the BP exercise for TS could be due to this exercise was not alternated with the SQ exercise in the same set, allowing to perform each set of the BP exercise with practically no residual fatigue (Weakley et al., 2020). In connection with the above, another noteworthy aspect was that participants of the TS group performed significantly ( *p* = 0.049−0.016) more repetitions and at faster velocities than those completed by the AS group in the SQ exercise during the entire training period (Tables 1 and 3). Again, this fact could be explained because of the lower degree of fatigue induced in the TS group by not interspersing the BP exercise during the inter-set recovery intervals of the SQ exercise. However, apparently, that difference had no advantageous impact on the performance improvement in the SQ exercise for TS compared to the AS group, since the gains obtained in the predicted 1RM, AV, AV ≥1, and AV<1 were practically identical for both groups (Table 5).

Regarding muscular endurance performance in the SQ exercise, none of the two experimental groups showed significant pre-post changes, although the AS group resulted in greater percent change and ES than the TS group. The high inter-subject variability likely observed in both experimental groups in the average MPV in the fatigue test after the training period has prevented finding statistically significant changes in this variable. Thus, despite the positive percentage of change observed in the AS group (+10.2%) and showing a significant “time x group” interaction concerning the TS group, it appears difficult to generalize and reach definite conclusions about which training set configuration improves muscular endurance the most.

Finally, both groups exhibited small and similar increments in CMJ performance (Table 5). These results are substantially lower (Δ: ∼3%; ES: 0.18−0.23) than those shown by previous studies (Δ: 5.3–12.3%; ES: 0.45−0.84) using a VBT approach with similar load magnitudes (50–70% 1RM) and %VL (10–20%) in the set (Rodriguez-Rosell et al., 2021b; Rodriguez-Rosell et al., 2021a). However, most of these studies carried out a longer training program (7–8 *vs.* 6-week duration) 31 or greater training frequency (three *vs.* two sessions per week) (Pareja-Blanco et al., 2017b) compared to the present study, which could favor greater changes in vertical jump capacity. Additionally, it is important to note that in these previous studies the training program was composed only of the SQ exercise, thus likely achieving a lower degree of fatigue in each training session.

The effects of consecutive completion of two different exercises targeting the same or antagonist muscle groups (also called super-set and paired set training), followed by a recovery period, have been previously addressed (Robbins et al., 2010c; Robbins, Young & Behm, 2010; Robbins et al., 2010b). However, the lack of longitudinal studies examining upper- and lower-limb exercise pairings (*e.g.*, SQ and BP) on athletic performance prevents a direct comparison with our results. Considering the results of our study, it seems that the presumed fatigue magnification induced by the AS configuration during each training session did not negatively affect upper- or lower-body strength development. These findings agree with a previous study showing that a circuit training (three single-joint exercises grouping for the upper- and lower-body) did not negatively affect strength adaptations compared to a traditional structuring of the same exercises, execution order, and training load (Alcaraz et al., 2011).

According to our results, if two resistance exercises involving different body segments (*i.e.,* upper- and lower-body muscle groups) are performed using an AS configuration achieving a moderate to low degree of fatigue in each set (*i.e.,* <20% VL) the improvement in athletic performance should not be significantly impaired compared to a traditional approach following a training program of several weeks.

The small sample size and the moderate strength level of the participants should be considered as the main limitation of the present research. Another possible limitation of this study is related to the training intervention duration, as it is possible that a training period longer than 6 weeks (12 sessions) could yield larger outcomes and/or differences between training conditions. Finally, the findings of the present study are limited to the population analyzed, and the specific training program performed. Therefore, despite the significance of the results obtained, it is worth noting that the current results may vary, for instance, with different exercise pairings and/or execution order, different relative loads and/or distribution along the training period, or a higher level of fatigue (%VL) incurred in the set. Thus, further studies are needed to clarify whether the observed effects would be similar under different training schemes (*e.g.*, exercises and training loads).

## Conclusions

Training programs using traditional or alternating set configurations combining SQ and BP exercises with moderate loads (55–70% 1RM) and achieving a low degree of fatigue in the set (15–20% VL) in both exercises, resulted in similar jump and strength improvements. However, AS showed a more time-efficient training strategy because it roughly halves that for the TS condition. A reduction of close to 50% in training time per session could be hugely beneficial for any practitioner, especially for those with time constraints to perform sessions using upper- and lower-body exercises.

##  Supplemental Information

10.7717/peerj.14636/supp-1 Supplemental Information 1 Values of pre-post neuromuscular changes

10.7717/peerj.14636/supp-2 Supplemental Information 2 Values of the training program performed

10.7717/peerj.14636/supp-3 Supplemental Information 3 Codebook

## Additional Information and Declarations

### Data Availability

The following information was supplied regarding data availability: The raw measurements are available in the Supplemental Files.
