# Supplementary material for: Upper-lower body super-sets vs. traditional sets for inducing chronic athletic performance improvements
Source: PeerJ. 2023 Feb 21;11:e14636. doi: 10.7717/peerj.14636 (PMC9951798; doi:10.7717/peerj.14636)
Supplement: Supplemental Information 3 [file peerj-11-14636-s003.docx]

| Acronym | Meaning |
| --- | --- |
| AV | average MPV attained against all absolute loads common to pre-and post-tests in the squat progressive loading test |
| AV ≥ 1 | average MPV attained against common loads that were lifted equal or faster than 1.00 m·s^−1^ in the squat progressive loading test |
| AV < 1 | average MPV against common loads lifted slower than 1.00 m·s^−1^ in the squat progressive loading test |
| AV ≥ 0.8 | average MPV attained against common loads that were lifted equal or faster than 0.80 m·s^−1^ in the bench press progressive loading test |
| AV < 0.8 | average MPV against common loads lifted slower than 0.80 m·s^−1^ in the bench press progressive loading test |
| 1RM | One repetition maximum |
| %VL | Magnitude (percentage) of intra-set velocity loss |
| CMJ | Countermovement jump |
| SD | Standard deviation |
| BP | Bench press |
| Pre | initial assessment |
| Post | final assessment |
| SQ | Full squat |
| rep/reps | Repetición/repeticiones |
| AS | Alternating set experimental group |
| TS | Traditional set experimental group |
| MPV | mean propulsive velocity |
| MPV_best_ | mean propulsive velocity of the fastest (usually first) repetition in the first set |
